# An Electrical Conductivity Sensor for the Selective Determination of Soil Salinity

**DOI:** 10.3390/s24113296

**Published:** 2024-05-22

**Authors:** János Horváth, László Kátai, István Szabó, Péter Korzenszky

**Affiliations:** Institute of Technology, Hungarian University of Agriculture and Life Sciences, Páter K. 1, H-2100 Gödöllő, Hungary; katai.laszlo@uni-mate.hu (L.K.); szabo.istvan.prof@uni-mate.hu (I.S.); korzenszky.peter.emod@uni-mate.hu (P.K.)

**Keywords:** soil salinity, soil sensor, conductivity, variable frequency, proximal measurement

## Abstract

The measurement of electrical conductivity (EC) has long been a tool for understanding soil properties. Previous studies concluded that EC measurement is not an ion-selective method, but these papers did not address the measurement frequency. An experimental tool and method were developed for semi-factory conditions in a large-scale soil trough at the Institute of Technology of the Hungarian University of Agricultural and Life Sciences. A specially designed and built test apparatus mounted on the tractor’s three-point hitch was used as a measuring device. The wear-resistant steel elements of the measuring device were also the sensors for measuring EC. This paper describes the conditions of the measurement series, the measurement results, and our conclusions from the experiments with the soil sensor. Different characteristics were measured in soil moistened with K and Ca solutions at different concentrations. The EC values show an increasing tendency with increasing salt concentration, and we also found that the rate of change of EC is different for different solution ratios. Based on our measurements, we found that the best method to isolate concentration differences is to use the test frequency range 20 Hz–250 kHz.

## 1. Introduction

### 1.1. Conductivity in Soil

**Figure 1:**
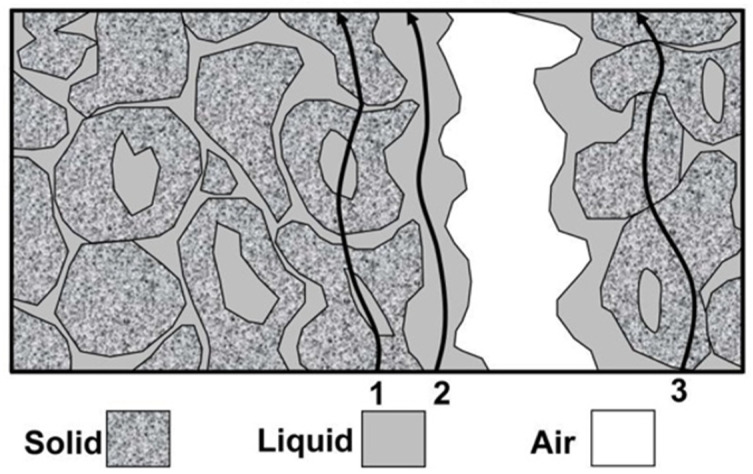
Direction of electrical conduction in three-phase soil [1,2].

The information obtained from measuring electrical conductivity (EC) is only approximate [1]. However, the use of EC measurements has seen a significant increase in recent years due to two reasons. Firstly, advancements in technology have made it possible to create smaller and more mobile conductivity-measuring devices. Secondly, satellite positioning systems have made conductivity an easy feature to measure. Figure 1 illustrates the possible directions of particle displacement during three-phase, unsaturated soil conductivity measurements. Three pathways of current flow contribute to the EC of a soil: (1) a liquid phase pathway via dissolved solids contained in the soil water occupying the large pores, (2) a solid–liquid phase pathway primarily via exchangeable cations associated with clay minerals, and (3) a solid pathway via soil particles that are in direct and continuous contact with one another. During this process, air acts as an insulating medium [1,2].

### 1.2. Chemical Composition of Soil Moisture in Soil Solution

Various inorganic salts, organic matter, and gases dissolve in soil moisture. Mineral salts that are dissolved dissociate into positively and negatively charged ions which are surrounded by a hydrate shell. The following ions are mainly present in the soil solution:

- Cations: Ca^2+^, Mg^2+^, Na^+^, K^+^, NH^+4^, and, in some soils, Al^3+^, Fe^3+^, or Fe^2+^.
- Anions: HCO^3−^, CO_3_^2−^, Cl^−^, SO_4_^2−^, NO^3−^, H_2_PO^4−^, HPO_4_^2−^.

Most dissolved organic compounds consist of organic acids and low molecular weight humic substances. Among dissolved gases, CO_2_ and O_2_ are the most noteworthy. Soluble substances found in soil primarily originate from weathering and soil formation processes. Nonetheless, salts infiltrate the soil solution from groundwater near the surface. Moreover, in agricultural regions, the salinity of the soil is altered by the introduction of fertilizers and compounds through irrigation water [3].

### 1.3. Hydrated Ions

The interaction between ions that are dissolved in water has been a topic of great interest due to its significance in various chemical, biological, and environmental processes [4]. Numerous experimental and theoretical studies have explored aqueous solutions of ions, both in the bulk [5,6] and at interfaces [7,8,9].

**Figure 2:**
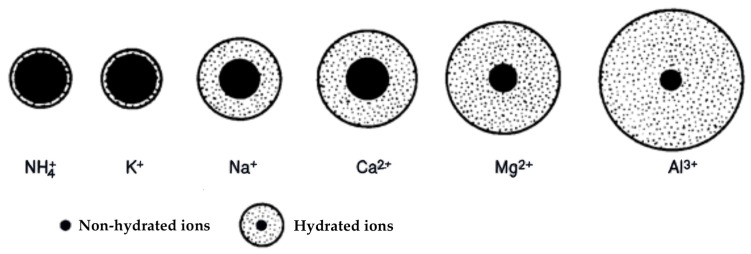
The size of the aqueous hydrate cover for each element in the soil [3].

Figure 2 illustrates the size of the hydrate shell in an aqueous solution. The thickness of the hydrate sphere in aqueous solution depends on the diameter of the dehydrated cation and the magnitude of its charge. The smaller the diameter of the dehydrated cation and the larger its charge is, the thicker the hydrate sphere becomes. This also influences the mobility of hydrated ions [3,10]. Therefore, it can be concluded that the size of the hydrate shell plays a vital role in understanding the behavior of ions in aqueous solutions.

**Figure 3:**
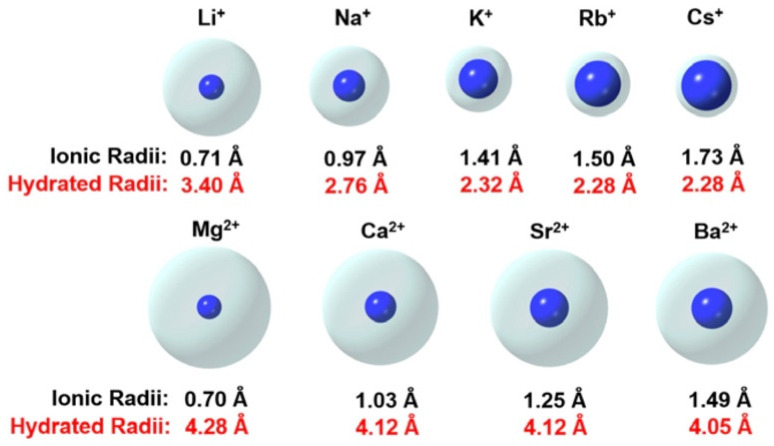
The effective sizes of ionic and hydrated radii of some of the common ions [13].

Common ions found in soil have a wide diversity of sizes [11], as depicted in Figure 3. As a result, sodium has more of a dispersing effect than potassium, and calcium has more of a flocculating effect than magnesium. These cations are traditionally called base or base-forming cations [12]. Sodium and calcium are easily exchanged in soils, while potassium and magnesium are incorporated permanently into mineral constituents. The composition of soils is heavily influenced by hydrate radiates.

### 1.4. Using Soil Sensors for EC Measurement

Nowadays, soil salinization is one of the leading issues, and it poses a risk to land productivity on a global scale [14]. Saline soils exist in over 100 countries and cover over 1.125 billion hectares of land all over the world [15]. This problem is growing at a rate of approximately 1–2% each year, and by 2050, it is estimated that soil salinity could potentially affect 50% of the available arable lands, which would be a significant obstacle to sustainable development in agriculture on a global scale [16].

Electrical conductivity (EC), as the reciprocal of resistance, is an important measurement tool for soil testing in precision farming practices. To understand the impedance of electrochemical objects, it is necessary to understand the behavior of simple electrical circuits, first in steady state, then in transient conditions. Such circuits contain simple linear electrical elements: resistance, capacitance, and inductance [17].

There is a large body of literature available on the development of EC sensors. Typically, these sensors have four electrodes [18,19,20], and the traditional current–voltage four-electrode method is designed to create an in-situ soil EC detector that is affordable, easy to operate, and has high measurement precision, as well as integral control and data processing procedures [20]. The proposed electrodes are biocompatible thanks to the absence of poisonous metals such as copper or nickel [21].

Apparent soil electrical conductivity is one of the simplest and least expensive soil measurements that can provide expedient information about soil characteristics and play a vital role in precision agriculture [19].

Soil sensors are becoming a popular solution for measuring relevant soil properties, such as nutrient content in real time, allowing farmers to obtain immediate information on the condition of the soil, which is the most important resource for production. This measurement method not only saves resources but also minimizes environmental impact by enabling judicious and site-specific application of nutrients. The use of soil sensors facilitates rapid, real-time, and cost-effective soil testing and nutrient mapping solutions. [22].

Understanding the correlation between soil EC and various soil properties in the field is crucial as it provides valuable insights into soil salinity, water content, texture, and structure. The data harvested from the soil can be converted into useful information and the information can be utilized in modern precision agriculture to support crop management and nutrient management decisions [23,24].

The range of frequencies commonly used for determining electrical conductivity (EC) in the field is typically between 100 Hz and a few hundred kHz. At lower frequencies, the polarization of the electrodes can interfere with the reading, and at higher frequencies (kilo- and megahertz), there can be a phenomenon called dispersion. Additionally, high-frequency conductivity meters can be expensive [23]. However, at frequencies below 1 MHz, capacitive and electrolytic effects on the measuring electrodes and the amplifier distortion can be avoided. This allows resistive effects to contribute predominantly to the signal, like unpolluted DC measurements [24,25,26].

Many electromagnetic induction (EMI) sensors work at frequencies lower than 300 kHz [27]. However, high-frequency electromagnetic induction (HFEMI) sensors operate in the range of 300 kHz to 30 MHz [28]. The idea behind taking measurements at different frequencies is that ions of various sizes exhibit varying conductivity with frequency changes. This principle provides the potential for selective detection.

This paper presents the fundamental research of EC measurement methodology through a self-developed EC soil sensor. The soil sensor is being developed in the Institute of Technology at the Hungarian University of Agriculture and Life Sciences. This methodology could replace the slower, more complex, and expensive laboratory determination of salinity with as many on-the-go measurements as possible.

## 2. Materials and Methods

### 2.1. The Goal of Measurement

**Figure 4:**
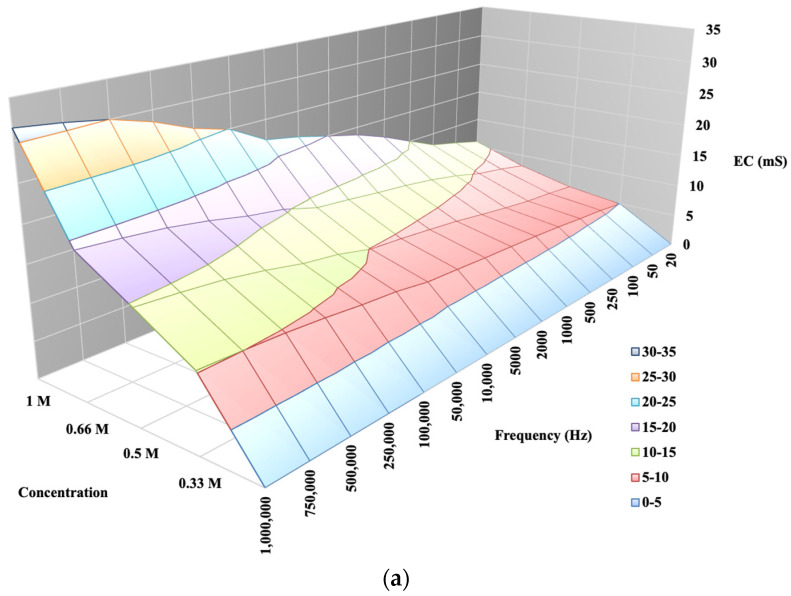

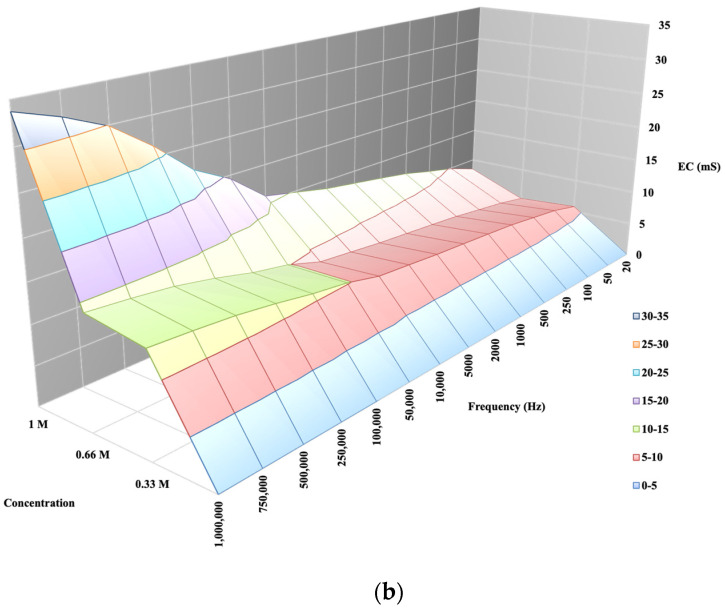
(**a**) EC and K concentration in soil and as a function of a signal sequence of known frequency [29]. (**b**) EC and Ca concentration in soil and as a function of a signal sequence of known frequency [30].

The laboratory model described by Horváth et al. in Figure 4a,b shows that in a known soil, by measuring its conductivity via varying the measurement frequency, there is a difference in the response functions of K and Ca soil solutions of the same concentration, i.e., for soil solutions containing the same concentration of cations, EC saturates according to different functions as a function of measurement frequency [29,30,31]. The aim of our measurements is to test whether the findings from a laboratory model can be confirmed under semi-factory conditions when measured on a real working apparatus, because this is the first step towards the analytical application of conductometry for the determination of the selective salinity of the soil.

Therefore, solutions of known composition at different known concentrations were mixed and the EC response functions were investigated using an LCR instrument by varying the measuring current frequency in a predetermined sequence.

### 2.2. The Subject of Measurement

The experiments were conducted at the laboratory of the Institute of Technology at the Hungarian University of Agriculture and Life Sciences at a constant temperature of 22 ℃. The aim of the measurement was to analyze the effect of cations of dissolved salts on soil EC under natural conditions. We chose K^+^ and Ca^2+^ as the selected cations because replacing these microelements in cultivated plants is an expensive issue. To introduce these ions into the soil solution, we used water-soluble salts in the form of KCl and CaCl_2_.

During the preliminary experiment, soil measured on a scale was poured into a mixing device first, followed by water. After mixing, we took a sample of the material and added soil and water until we finally had a material with a structure that still holds water. As a result, 7 kg of water had to be added to 31 kg of soil so that one portion was available. After determining the mixing ratio of soil and water, the concentrations of the tested samples were determined. During the test, we worked with several solutions with different molar concentrations. As a starting point, we calculated the mass of 1 mol (1 M KCl = 74.55 g; 1 M CaCl_2_ = 110.98 g). We determined the amount of raw material required for mixing per 0.1 mol. After that, we determined how many grams of raw material we needed for 7 kg of water for our dilution series. A dilution series of 1 M, 0.66 M, 0.5 M, 0.33 M, and distilled water was prepared, where 0 M served as a reference measurement. Table 1 shows the amounts of KCl and CaCl_2_ corresponding to the dilution series for 7 kg of water.

**Figure 5:**
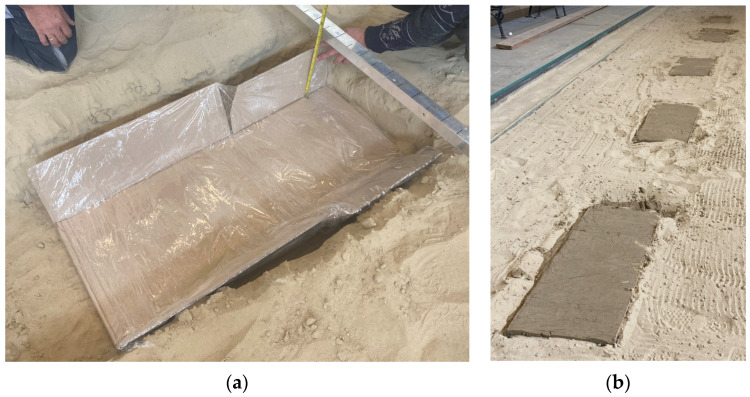
Preparing the measuring pit for measurement (**a**) and the filled pits (**b**).

Based on these, the recipe needed to mix one dose was available to us. Later, five mixings were necessary to produce soil paste of the appropriate quantity. The measurements were carried out in a 30 m long sand-filled demo track in the Institute of Technology’s laboratory in a closed building. In the demo track, five brick-body shaped measuring pits were constructed with the following dimensions: 1 m × 0.45 m × 0.15 m. The goal was to make three measurements on each section so that the soil sensor could be placed in each case. The pits were isolated from their surroundings on all sides to prevent leakage of the solution applied and each pit was filled with the original sand saturated with solutions prepared in known concentrations as shown in Figure 5.

### 2.3. The Soil Sensor and the LCR Meter

**Figure 6:**
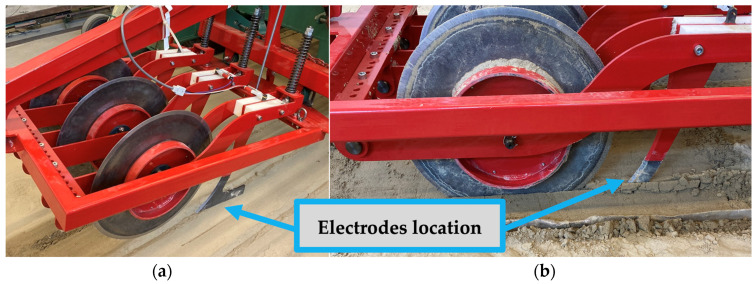
Power tillage implement front mounted on three-point hitch, consisting of three working elements in the hold-on position (**a**) and coulters in action (**b**).

The measuring device used in our experiments was a pushed system sensor developed by the Institute of Technology, which was mounted on the standard front three-point hitch of the tractor and consisted of three operating elements as shown in Figure 6 [32]. The soil sensor was equipped with wear-resistant steel in-line choroidal columns that acted as sensors. The distance of the spacer could be adjusted between 200 and 500 mm in steps of 25 mm, and they were separated from the frame structure by a polyamide sleeve. For our measurements, we set the distance of the spacers to 250 mm and the working depth to 95 mm.

In front of the sensors, ground-driven cutting discs worked to prepare the soil for measurement by cutting its upper layer. The same discs also cut the crop residues, reducing their impact on the measurement results. The depth of penetration into the soil was ensured by the two-sided rim of the discs and the independently operating kicking mechanism. The link between the measuring device and the tractor front three-point linkage was provided by the frame structure. The measuring frame was self-aligning around the vertical axis within an angular range of ±30°, thus following the tractor’s direction of travel in the pushed position and ensuring that both sides of the sensors were in contact with the ground even when changing direction.

**Figure 7:**
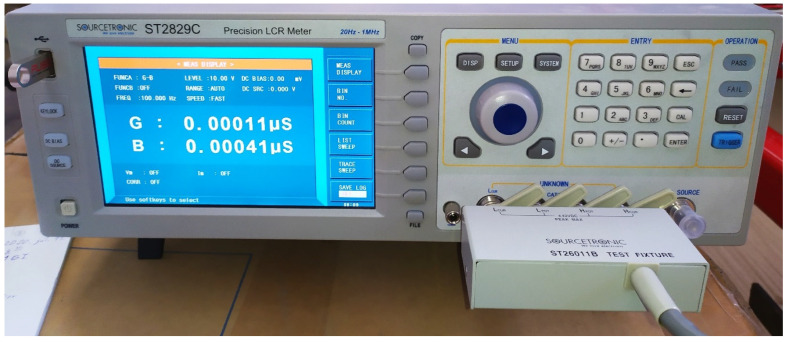
Sourcetronic ST2829C LCR meter.

The measurements were made with a Sourcetronic High Precision ST2829C LCR meter (Manufacturer: Sourcetronic GmbH, Bremen, Germany), as shown in Figure 7, mounted on the measuring trolley. The LCR meter is an instrument used to measure the inductance (L), capacitance (C), and resistance (R) of a component. It has a resolution of 0.00001 nS and a basic accuracy of 0.05%. The measurement was performed with an electrode voltage of 10 V and a measurement frequency range of 20 Hz to 250 kHz: 20, 100, 250, 1000, 10,000, 100,000, 250,000 Hz. The output impedance of the instrument was set to 100 Ω.

The principle of operation of the LCR measuring instrument is to provide a constant excitation at its output through a four-wire measuring system and to detect the response function from the ground sensor. The measuring data logger emits a signal sequence of known frequency and amplitude and measures the response function from the cultivation element. The conductance G (Siemens, S) and susceptance B (Siemens, S) are recorded in a file.

**Figure 8:**
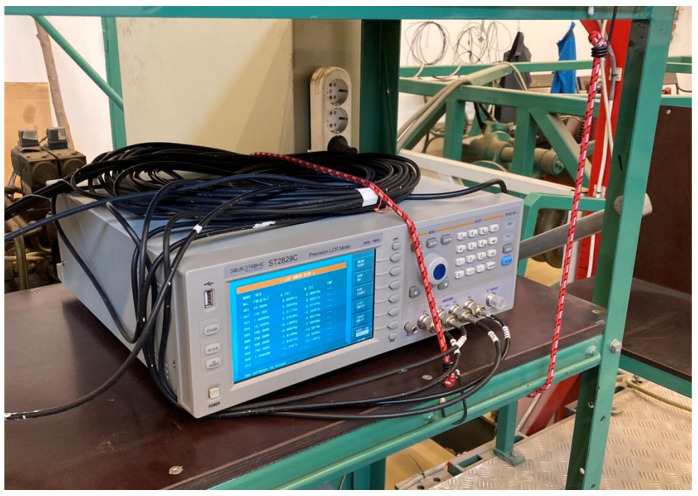
The instrument shelf.

The laboratory LCR meter was placed on the instrument shelf (Figure 8) of the sensor-carrying trolley, where we could provide a vibration-free environment for the instrument while in motion.

**Figure 9:**
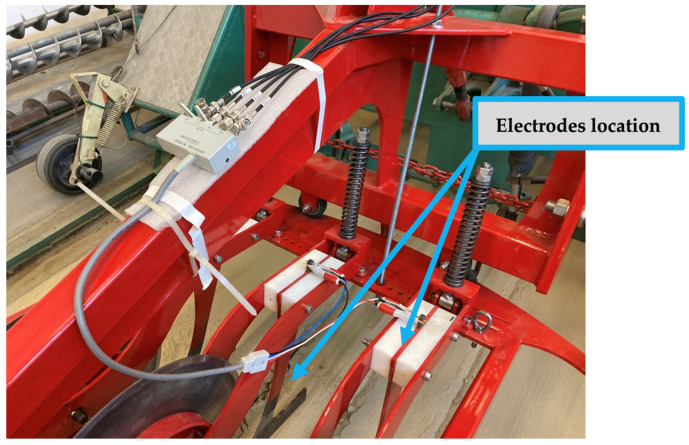
Wiring of the Sourcetronic ST2829C precision LCR meter.

When wiring the LCR meter, the characteristics of the sensor were considered and a stable wiring of the laboratory instrument was achieved, even during movement of the sensor, as shown in Figure 9 below.

### 2.4. The Measurement and Data Processing Process

**Figure 10:**
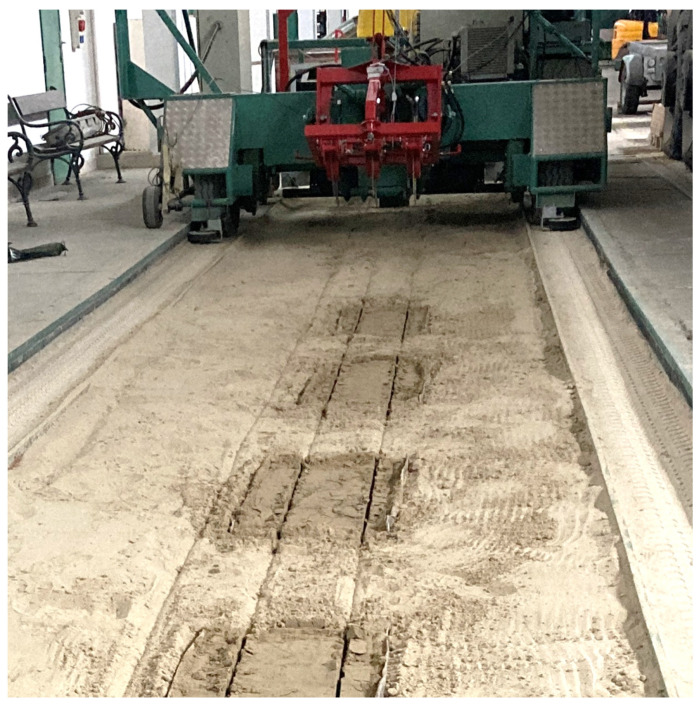
The soil sensor in the soil trough after measurement.

In our electrical conductivity experiments, the measuring trolley was manually stopped, and measurements were taken at each measuring pit for 1/1/1 min, in the beginning/middle/end of the pit. The results of the measurement process are shown in Figure 10 below, with the soil probe cultivation traces.

At the measurement points, the LCR meter performed the measurements at the pre-programmed frequencies, and then repeated the measurement process at the end of the sequence if the probe was in the soil solution. The measured data were saved to USB memory and loaded into Microsoft Excel for Mac, Version 16.85 (24051214). For each measurement at each soil solution concentration, the median of the data series was calculated for each signal sequence of known frequency and plotted in Excel. The measurement results are presented in the next Results section.

## 3. Results and Discussion

To detect the cation content of single-phase soil solutions, the typical reference G saturation curves were determined by measurements with predetermined solution concentrations in the process described in the Materials and Methods section.

### 3.1. Determination of Response Functions for Soil Solutions Containing Potassium Cations

**Figure 11:**
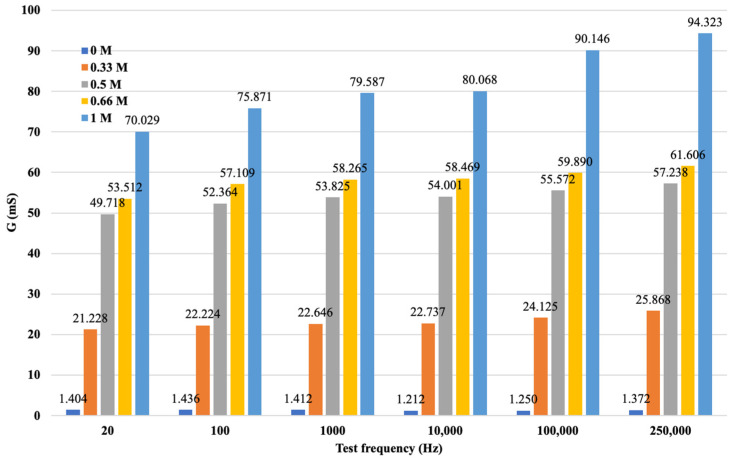
G values for soil solutions with different concentrations of KCl.

After data cleaning and processing, it was possible to analyze the processed data and record the measurement results by cation, concentration, and varying measuring frequencies. The results of the processing are illustrated in Figure 11.

**Figure 12:**
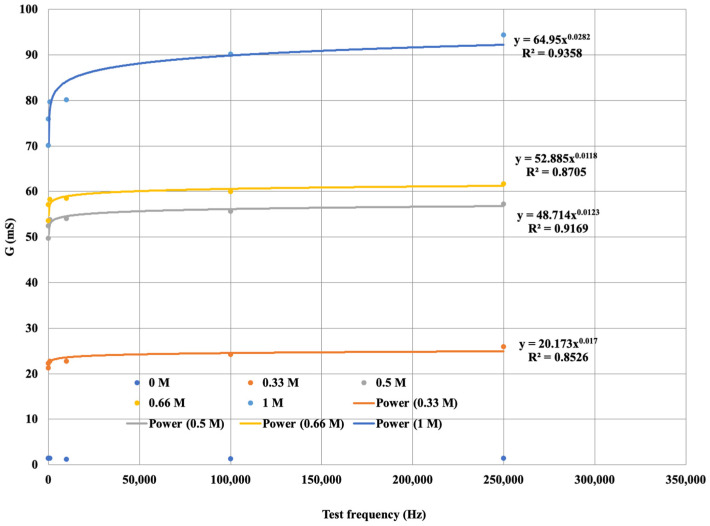
G saturation curve for soil solutions with different K^+^ concentrations.

The diagram is more visible if the variation in the electrical conductivity of the solutions tested is plotted as a function of frequency. Figure 10 not only shows that the conductance G increases with increasing concentration in a function, but also that G increases with increasing frequency. From the diagram for the same concentration of solution as the signal series, with increasing frequency, the measurement output, i.e., G, also increases. The higher the ionic concentration of the solution, i.e., the saltier the solution, the higher the value of G that can be calculated from the response function. We were able to fit the resulting points to strictly monotonic increasing power functions with a coefficient of determination R^2^ > 0.85, i.e., a close fit. This gives the response functions for the different K^+^ cation concentrations. The functions are presented in Figure 12, where only the measured values are plotted for the neutral (0 M) soil solution taken as a reference measurement and the fitted regression line is not plotted.

Thus, the potassium (K^+^) cation reference functions for the measurement model set-up show a close fit.

### 3.2. Determination of Response Functions for Calcium Cation Soil Solutions

**Figure 13:**
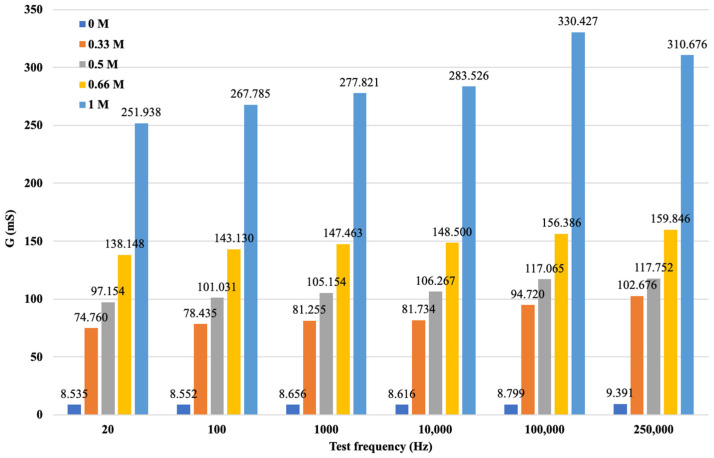
G values for soil solutions with different concentrations of CaCl_2_.

The same calculation method was followed to generate the data series as previously described for the potassium measurements. Also, for the calcium solution measurements, frequency-dependent arithmetic averages of the total measurement were determined. After the calculations were performed, it was possible to record the measurement results of the calculated data series by ion, concentration, and different measuring frequencies, summarized in Figure 13.

**Figure 14:**
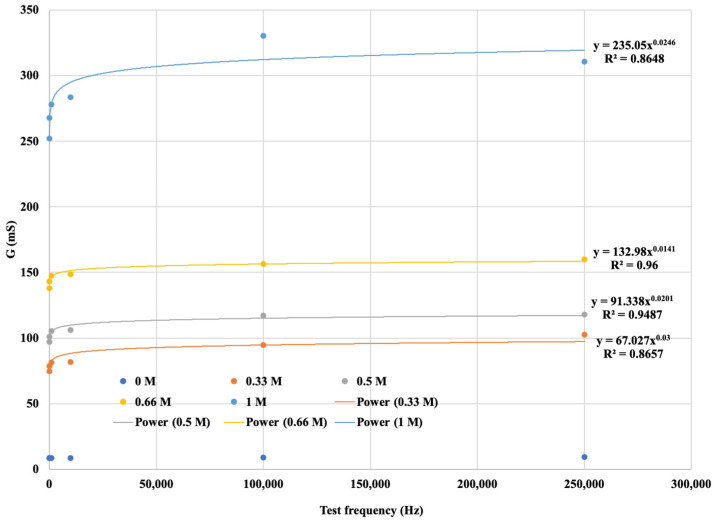
The G saturation curve for soil solutions with different Ca^2+^ concentrations.

The diagram is more informative if the variation in the electrical conductivity of the solutions tested is plotted as a function of frequency. Figure 12 shows not only that G increases with increasing concentration in a function, but also that when the signal series increases in frequency, the G value still increases, and that it is the kHz frequencies that seem to be suitable for detecting Ca^2+^ content at higher concentrations. The function shows that for the same solution concentration, the measurement output, i.e., the G value, increases in proportion to the increase in a signal sequence of known frequency. Furthermore, the higher the ionic concentration of the solution, the saltier the solution, and the higher the G value calculated from the response function. The resulting points are fitted with strictly monotonic increasing power functions with coefficient of determination R^2^ > 0.86, i.e., a close fit. This gives the reference functions for the different Ca^2+^ cation concentrations. The functions are shown in Figure 14, where the measured values are plotted for the neutral (0 M) soil solution taken as reference measurement cask the fitted regression line is not plotted.

### 3.3. Comparison of Potassium and Calcium Reference Functions

A comparison of the reference functions is presented in Table 2 below.

Our results show that by selectively measuring the electrical conductivity of a soil with a known electrolyte concentration at the same time as a reasonable change in the measuring current frequency, the response function G will vary from electrolyte to electrolyte. By comparing the response functions at different concentrations of the solutions, we find that the responses of the solutions to varying the measurement frequency display different behaviors because the exponents of the strictly monotonic increasing power functions differ from each concentration pair. The difference in exponents explains the different behaviors of the cations.

## 4. Conclusions

In this article, a test apparatus was presented as well as a measurement method to investigate the salinity of soils using the principle of EC measurement, applying a series of signals with different measurement frequencies.

Our measurements show that, by selectively measuring the electrical conductivity of a soil paste enriched with a known electrolyte concentration at the same time as a reasonable change in the measuring current frequency, the response function G will vary from electrolyte to electrolyte. By comparing the response functions at different concentrations of the solutions, it has been found that the responses of the solutions to varying the measurement frequency exhibit different behaviors because the exponents of the strictly monotonic increasing power functions differ from concentration pair to concentration pair. The difference in exponents explains the different behaviors of the cations. Thus, potassium and calcium solutions of the same concentration in the same environment with the same measurement parameters behave differently because the size of the hydrated cations differs significantly, so that they move at different rates for a given electric field in the same viscous medium.

Our experiments proved a relationship between soil salt concentration, a signal sequence of known frequency, and measured EC that can be described by a function. By increasing the frequency, the measured EC value of the soil saturated with solutions of a given concentration changed according to the functions shown in Figure 12 and Figure 14, so saturating the soil with solutions containing the same concentrations of cations (K^+^ and Ca^2+^) gives different EC functions depending on signal sequence of known frequency.

Furthermore, the experimental results on K^+^ and Ca^2+^ cations provide an opportunity to extend the established relationships to other elements of the liotropic series, such as Mg^2+^ and Na^+^ cations and cation combinations, by further investigations. To create a practical, so-called “black box”, i.e., a measurement method, we consider it worthwhile to develop calibration functions for several soil types with our method.

## Figures and Tables

**Table 1:** The amounts of KCl and CaCl2 in the case of 7 kg of water.

Concentration	KCl (g)	CaCl_2_ (g)
1 M	521.85	776.86
0.66 M	344.42	512.72
0.5 M	260.92	388.43
0.33 M	172.21	256.36
0 M	0	0

**Table 2:** Comparison of reference functions.

Concentration	K^+^	Ca^2+^
1 M	f(x) = 64.95x^0.0282^	f(x) = 235.05x^0.0246^
0.66 M	f(x) = 52.885x^0.0118^	f(x) = 132.98x^0.0141^
0.5 M	f(x) = 48.714x^0.0123^	f(x) = 91.338x^0.0201^
0.33 M	f(x) = 20.173x^0.017^	f(x) = 67.027x^0.03^

## Data Availability

The data presented in this study are available on request from the corresponding author.
